# Prevalence of Albuminuria in Cardiology and Endocrinology Departments and Its Influencing Factors: A Multicenter, Real-World Evidence Study in China

**DOI:** 10.1155/2020/1231593

**Published:** 2020-05-02

**Authors:** Qian Ren, Changsheng Ma, Jiguang Wang, Xiaohui Guo, Linong Ji

**Affiliations:** ^1^Department of Endocrinology and Metabolism, Peking University People's Hospital, Beijing, China; ^2^Department of Cardiology, Beijing Anzhen Hospital, Capital Medical University, Beijing, China; ^3^Centre for Epidemiological Studies and Clinical Trials, Shanghai Key Laboratory of Hypertension, The Shanghai Institute of Hypertension, Department of Hypertension, Ruijin Hospital, Shanghai Jiaotong University School of Medicine, Shanghai, China; ^4^Department of Endocrinology, Peking University First Hospital, Beijing, China

## Abstract

**Aims:**

To evaluate the prevalence of albuminuria and compare its risk factors in diabetic and hypertensive patients.

**Methods:**

This was an observational, cross-sectional, multicenter registry across China. Consecutive patients were registered with the Cardiology and Endocrine departments in 40 centers. Clinical characteristics were collected, and urinary albumin-to-creatinine ratio (UACR) was measured using the immunochemical method.

**Results:**

Of the 2510 patients enrolled in the study, 1515 underwent UACR testing and were included in the present analysis. The prevalence of microalbuminuria was 13.0% and 16.1% while that of macroalbuminuria was 2.5% and 5.0%, in the Cardiology and Endocrinology departments, respectively. HbA1c and systolic blood pressure (SBP) were independent risk factors for albuminuria. The relationship of blood pressure (BP) and HbA1c with albuminuria was continuous and graded. Compared with the reference level of SBP 130–139 mm Hg, an SBP level of <130 mmHg was significantly associated with a lower risk of albuminuria in all subjects (OR = 0.60; 95% CI: 0.40–0.89; *P* < 0.001) and in subjects with concomitant hypertension and diabetes (OR = 0.48; 95% CI: 0.25–0.92; *P* < 0.001).

**Conclusions:**

In China, nearly one-fifth of patients in the Cardiology and Endocrinology departments have albuminuria although ACEI/ARB were widely used. More effective therapy is needed in this population.

## 1. Introduction

Albuminuria, both microalbuminuria and microalbuminuria, are important indexes in clinical practice. First, in diabetes management, microalbuminuria is one of the earliest evidences of diabetic nephropathy [1]. Besides, the treatment guidelines from the American Diabetes Association for the management of diabetes pointed out that albuminuria was also a risk factor for cardiovascular diseases in all patients with diabetes [2]. Second, in hypertension management, it is demonstrated that microalbuminuria is also a prognostic marker for cardiovascular diseases, and for renal insufficiency, and all-cause mortality in patients with hypertension [3–5].

Therefore, screening for albuminuria is of great importance in the Endocrinology and Cardiology departments. According to the Prevention of Renal and Vascular End-stage Disease (PREVEND) study, which involved 40,856 inhabitants of Groningen, the Netherlands [6], the prevalence of microalbuminuria was 6.6% (excluding diabetic and hypertension patients) in the general population. In the diabetic population, I-DEMAND (Italy Developing Education and awareness on MicroAlbuminuria in patients with hyperteNsive Disease) study, which was an observational survey held in 87 centers of specialized care, showed that the prevalence of microalbuminuria was 37% in diabetic patients [7].

In the hypertensive population, two large-scale population surveys, the NHANES III [8] and PREVEND study [6], showed that the prevalence of microalbuminuria was 16% and 11.5%, respectively. In hypertensive outpatients attending a cardiologist or internist, a population at a relatively high risk of cardiovascular disease, i-SEARCH (Survey for Evaluating Microalbuminuria Routinely by Cardiologists in patients with Hypertension) reported a very high prevalence of 58.4% microalbuminuria [9]. However, this survey was published 10 years ago, using a semiquantitative test, and only patients from Taiwan district were included, which cannot represent the situation across China.

So, the first aim of our study was to evaluate the prevalence of albuminuria, especially microalbuminuria in a real-world design, multicenter registry in China, in patients with diabetes as well as in patients with hypertension.

In addition, previous studies have evaluated many influencing factors for albuminuria, such as age, gender, body mass index, a high-protein meal, vigorous exercise, smoking status, BP, blood glucose, hypercholesterolemia, genetic background, and metabolic syndrome [10–16]. Among them, BP and blood glucose received the most extensive and highly consistent body of evidence [7, 12, 17]. For example, in the PREVEND study, it was observed that microalbuminuria was independently related to hypertension and diabetes [6]. However, it is still not clear which of them plays a major role. Therefore, the second objective of our study was to compare the risk factors for albuminuria in a population of hypertensive and hyperglycemic patients.

## 2. Materials and Methods

### 2.1. Study Design and Participants

All the patients included in this study were from the ATTEND study, which was published previously [18]. To be brief, it was an observational, cross-sectional, multicenter registry study conducted in China from June 2011 to March 2012. Consecutive patients were registered with the Cardiology and Endocrine departments. The ethics committees of all participating hospitals approved the study protocol, and all subjects enrolled in the study gave the written informed consent before the initiation of the study.

### 2.2. Clinical and Biochemical Measurements

Baseline characteristics such as body weight and body height were measured in all subjects. Body mass index (BMI) was calculated as the body weight in kilograms divided by the body height in meters squared. BP was measured using a validated Omron HEM-7201 automatic oscillometric BP monitor (Omron Healthcare, Kyoto, Japan) at the first and second clinic visits. At both visits, three BP readings were obtained in the seated position after the patients had rested for at least 5 min. These six readings were averaged for statistical analysis.

Fasting plasma glucose (FPG) levels were measured using the glucose oxidase method. HbA1c was measured using a high-performance liquid chromatography (Ultra2 HbA1c Detector, PRIMUS Corporation, USA, normal range: 4–6%). The urinary routine test was performed on fresh urine samples, and the UACR was measured using the immunochemical method as described in the ATTEND study [18].

### 2.3. Statistical Methods

The basic statistics on study parameters were presented by number (%) and mean (SD). Student's *t*-test was used to compare continuous normally distributed variables between groups. A logistic regression analysis was used to calculate ORs, 95% CIs, and the corresponding *P* values. All statistical tests were performed using SAS software (version 9.13; SAS Institute, Cary, North Carolina, USA). A *P* value <0.05 was considered statistically significant.

## 3. Results

### 3.1. Characteristics of the Study Subjects and Prevalence of Albuminuria

A total of 2510 patients were enrolled in the ATTEND study. The exact type and percentages of antihypertensive treatments included angiotensin II receptor blockers (ARB) (37.8%), angiotensin enzyme inhibitor (ACEI) (19.6%), beta-adrenergic receptor blocker (29.3%), diuretics (9.0%), calcium channel blockers (CCB) (55.8%), compound antihypertensive drug (7.2%), *α*-receptor blocker (0.4%), antihypertensive drugs with central action (0.3%), and other antihypertensive drugs including traditional Chinese medicine (1.5%). Among them, 894 (69.3%) patients in the Cardiology department and 621 (53.2%) patients in the Endocrinology department underwent UACR testing and were included in the present analysis. The prevalence of microalbuminuria was 13.0% (116 patients) and 16.1% (100 patients), while the prevalence of macroalbuminuria was 2.5% (22 patients) and 5.0% (31 patients) in the Cardiology and Endocrinology departments, respectively.

Based on their test results, all the subjects were divided into three groups: patients with normal albuminuria (Normal group, *n* = 1246), patients with microalbuminuria (Micro group, *n* = 216), and patients with macroalbuminuria (Macro group, *n* = 53). The clinical characteristics of these subjects in the three groups are shown in Table 1. SBP was lowest in the Normal group and increased from the Micro group to the Macro group, and BP control rates were decreased from the Micro group to the Macro group. FPG and HbA1c levels were the lowest in the Normal group; the levels increased from the Micro group to those in the Macro group. Diabetes mellitus (DM) control rates decreased from the Micro group to those in the Macro group.

### 3.2. Risk Factors Associated with Albuminuria

A multivariate logistic analysis of albuminuria rate showed that HbA1c and SBP were independent risk factors for albuminuria in all subjects as shown in Table 2 (all *P* < 0.001). The relationship of BP and HbA1c with albuminuria is relatively continuous and graded. For example, in all subjects with concomitant hypertension and diabetes, HbA1c increased by 0.5%, the risk of albuminuria increased by 14% (*P* < 0.001), SBP increased by 5 mmHg, and the risk of albuminuria increased by 20% (*P* < 0.001).

We then analyzed the relationship between albuminuria rate and SBP and HbA1c by performing a univariate logistic analysis. A reference level of BP 130–140 mmHg was used, and the risk of albuminuria in the low-BP group (<130 mmHg) and the high-BP group (≥140 mmHg) was evaluated. Similarly, a reference level of HbA1c 6.5–7.0% and the risk of albuminuria in the low-HbA1c group (<6.5%) and the high-HbA1c group (≥7%) was analyzed. The risk of albuminuria in the BP ≥ 140 mmHg group reached 2.17 times as compared with the reference. The risk of albuminuria in the HbA1c ≥ 7% group was the highest and reached 3.17 times compared with reference as shown in Table 3.

**Figure 1:**
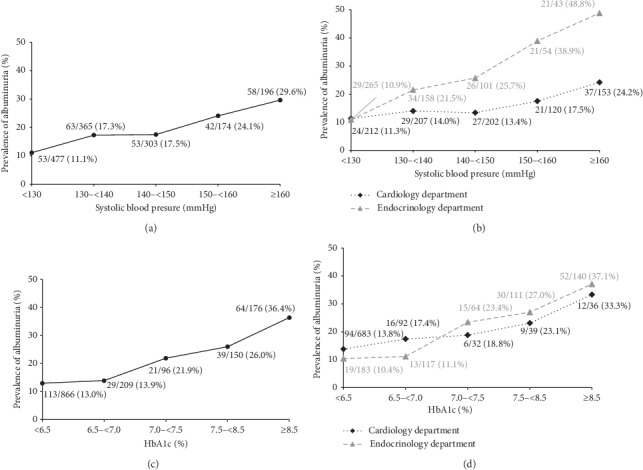
Prevalence of albuminuria in relation to SBP and HbA1c. (a) Prevalence of albuminuria in relation to SBP in all the subjects. (b) Prevalence of albuminuria in relation to SBP in subjects in the Cardiology department and Endocrinology department. (c) Prevalence of albuminuria in relation to HbA1c in all the subjects. (d) Prevalence of albuminuria in relation to HbA1c in subjects in the Cardiology department and Endocrinology department.

**Figure 2:**
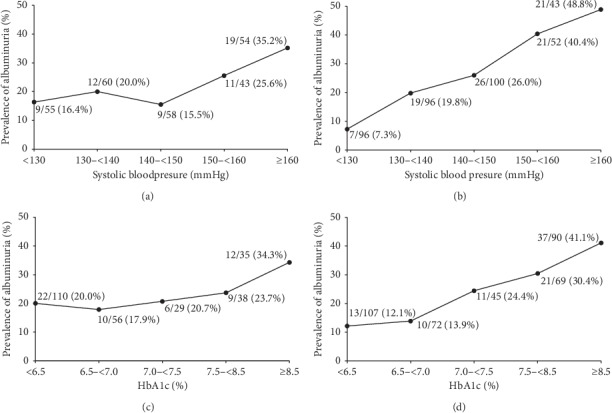
Prevalence of albuminuria in relation to SBP and HbA1c in subjects with concomitant hypertension and diabetes. (a) SBP in the Cardiology department. (b) SBP in the Endocrinology department. (c) HbA1c in the Cardiology department. (d) HbA1c in the Endocrinology department.

In a stratified analysis, we analyzed the prevalence of albuminuria in relation to SBP and HbA1c in all the participants as well as in the Cardiology and Endocrinology departments separately. The results are presented in Figure 1. With the increase of SBP from less than 130 mmHg to higher than 160 mmHg, the prevalence of albuminuria increased from 11.1% to 29.6%. Also, with the increase of HbA1c from less than 6.5% to higher than 8.5%, the prevalence of albuminuria increased even higher, that is, from 13.0% to 36.4%. Then, we analyzed the prevalence of albuminuria in relation to SBP and HbA1c in subjects with concomitant hypertension and diabetes in the Cardiology and Endocrinology departments as shown in Figure 2. Even SBP was at the same level; patients in the Endocrinology department showed a higher prevalence of albuminuria than those in the Cardiology department as shown in Figures 2(a) and 2(b). However, the effect of HbA1c was totally different. At the same level of HbA1c less than 7%, the patients in the Endocrinology department showed a lower rate of albuminuria than those in the Cardiology department, while at the same level of HbA1c higher than 7%, the patients in the Endocrinology department showed a higher rate of albuminuria than those in the Cardiology department as shown in Figures 2(c) and 2(d).

## 4. Discussion

In this real-world, multicenter registry study in China, we found that [1] the prevalence of microalbuminuria was 13.0% and 16.1% while that of macroalbuminuria was 2.5% and 5.0% in the Cardiology department and the Endocrinology department, respectively [2]. HbA1c and SBP were independent risk factors for albuminuria, and the relationship of BP and HbA1c with albuminuria was relatively continuous and graded, and [3] HbA1c played a major part in relationship to albuminuria.

In this study, the prevalence of microalbuminuria in hypertensive patients was found to be 13.0%, similar to the two large-scale population surveys, namely, the NHANES III study (16%) [8] and the PREVEND study (11.5%) [6], but much lower than that reported in the i-SEARCH study (58.4%), which was a clinic-based survey [9]. Similarly, in diabetic patients, the prevalence of microalbuminuria (16.1%) was still much lower than that in the I-DEMAND study (37%), which was also a clinic-based survey [7]. The possible reason for this may be because of the selection bias and the widespread uses of ACEI and ARB nowadays. In this real-world study, a total of 57.4% of patients were treated with ACEI (19.6%) or ARB (37.8%). Recently, new evidences suggest that SGLT2 inhibitors and GLP-1 agonists have renoprotective effects [19]. And by 2017, SGLT2 inhibitors and GLP-1 agonists had comprised 17% and 11% of new first- to fourth-line prescriptions [20]. So, the prevalence of albuminuria may change in the next decade.

Our findings that HbA1c and SBP were independent risk factors for albuminuria were in line with the results of previous studies [7, 12, 17, 21]. Further, according to the different evidence-based guidelines, antihypertensive treatments should consider factors such as frailty, comorbidities, and albuminuria especially in patients aged above 70 years [22, 23]. The relationship of BP and HbA1c with albuminuria is relatively continuous and graded. For example, in all subjects with concomitant hypertension and diabetes, HbA1c increased by 0.5%, the risk of albuminuria increased by 14% (*P* < 0.001), SBP increased by 5 mmHg, and the risk of albuminuria increased by 20% (*P* < 0.001). More importantly, we also found that compared with the reference level of SBP 130–140 mmHg, an alternative level of less than 130 mmHg could significantly lower the risk of albuminuria by 40% (OR = 0.60; 95% CI: 0.40–0.89; *P* < 0.001) in all subjects and lower the risk of albuminuria by 52% (OR = 0.48; 95% CI: 0.25–0.92; *P* < 0.001) in subjects with concomitant hypertension and diabetes. This finding added more evidences for the new definition of high BP (≥130/80 mmHg) in 2017 High Blood Pressure Clinical Practice Guideline [24].

We also compared risk factors for albuminuria in a population of hypertensive patients with hyperglycemic patients. In the Endocrinology department, the influence of SBP on albuminuria was more than that in the Cardiology department. SBP in the Endocrinology department was not so high, but all patients had a history of significant hyperglycemia (7.57 ± 1.77% and 6.15 ± 0.99% in the Endocrinology and Cardiology departments, respectively, *P* < 0.001) [18]. This history of hyperglycemia aggravated the influence of SBP at a relatively lower BP level on albuminuria in the Endocrinology department as shown in Figures 2(a) and 2(b). However, in the Cardiology department, the level of SBP was much higher than that in the Endocrinology department (141 ± 16.9 mmHg vs. 132.3 ± 17.0 mmHg; *P* < 0.001) [18]. However, the history of hypertension in the Cardiology department did not worsen the influence of hyperglycemia on albuminuria in the Cardiology department as shown in Figures 2(c) and 2(d). So, HbA1c might play a major role in relationship to albuminuria.

## 5. Conclusions

To conclude, in China, nearly one-fifth of patients in the Cardiology and Endocrinology departments have albuminuria although ACEI/ARB were widely used. HbA1c and SBP were independent risk factors for albuminuria, and the relationship of BP and HbA1c with albuminuria is relatively continuous and graded. More effective therapy is needed in patients with albuminuria.

## Figures and Tables

**Table 1:** Summary of demographic and disease characteristics in the three groups.

	Normal group	Micro group	Macro group
No. (male/female)	1246 (564/682)	216 (100/116)	53 (31/22)
Age (years), mean (SD)	58.0 ± 11.9	59.9 ± 11.9	57.6 ± 13.3
BMI (kg/m^2^), mean (SD)	25.3 ± 3.5	25.5 ± 3.8	26.3 ± 3.8
SBP (mmHg), mean (SD)	137 ± 17	143 ± 18	153 ± 20
FPG (mmol/L), mean (SD)	6.69 ± 2.41	7.75 ± 2.91	8.39 ± 3.78
HbA1c (%), mean (SD)	6.5 ± 1.4	7.3 ± 2.0	7.6 ± 2.1
Metabolism syndrome, *n* (%)	731 (58.7)	146 (67.6)	37 (69.8)
Ischemic heart disease, *n* (%)	161 (13.1)	37 (17.1)	9 (17.0)
Myocardial infarction, *n* (%)	32 (2.6)	6 (2.8)	0
Stroke, *n* (%)	68 (5.5)	10 (4.6)	2 (3.8)
BP control, *n* (%)	672 (53.9)	94 (43.5)	12 (22.6)
DM control, *n* (%)	951 (76.3)	122 (56.5)	24 (45.3)

**Table 2:** Multivariate logistic analysis of influencing factors for albuminuria rate.

Population	Clinical characteristics	Alternative level	Reference level	OR (95% CI)^‡^	*P* value^§^
All subjects with UACR results	Gender	Female	Male	0.76 (0.57, 1.01)	0.060
	HbA1c (%)	Increase by 0.5		1.16 (1.11, 1.21)	<0.001
	MS^¶^	Yes	No	1.31 (0.97, 1.77)	0.077
	SBP (mmHg)	Increase by 5		1.14 (1.10, 1.19)	<0.001
All subjects with concomitant high BP and DM	Gender	Female	Male	0.71 (0.48, 1.06)	0.093
	HbA1c (%)	Increase by 0.5		1.14 (1.08, 1.21)	<0.001
	MS	Yes	No	1.51 (0.93, 2.47)	0.099
	SBP (mmHg)	Increase by 5		1.20 (1.13, 1.27)	<0.001

^†^The step forward method is utilized with entry alpha = 0.2 as the selection criterion of the covariates.^‡^OR < 1 favors alternative level. CIs are Wald CIs. ^§^*P* values are based on Wald chi-square tests. ^¶^MS, metabolic syndrome.

**Table 3:** Univariate logistic analysis of albuminuria rate with SBP and HbA1c.

Population	SBP				HbA1c			
	Alternative level	Reference level	OR^†^ (95% CI)	*P* value^‡^	Alternative level	Reference level	OR^†^ (95% CI)	*P* value^‡^
All subjects with UACR	<130	130–<140	0.60 (0.40, 0.89)	<0.001	<6.5	6.5–<7.0	0.93 (0.60, 1.45)	<0.001
	≥140	130–<140	1.41 (1.02, 1.95)	<0.001	≥7.0	6.5–<7.0	2.58 (1.66, 4.03)	<0.001
Subjects with concomitant hypertension and diabetes	<130	130–<140	0.48 (0.25, 0.92)	<0.001	<6.5	6.5–<7.0	1.04 (0.57, 1.89)	0.071
	≥140	130–<140	1.78 (1.13, 2.80)	<0.001	≥7.0	6.5–<7.0	2.47 (1.45, 4.21)	<0.001
Cardiology subjects with concomitant hypertension and diabetes	<130	130–<140	0.78 (0.30, 2.03)	0.337	<6.5	6.5–<7.0	1.15 (0.50, 2.63)	0.722
	≥140	130–<140	1.34 (0.65, 2.79)	0.171	≥7.0	6.5–<7.0	1.66 (0.73, 3.73)	0.159
Endocrinology subjects with concomitant hypertension and diabetes	<130	130–<140	0.32 (0.13, 0.80)	<.001	<6.5	6.5–<7.0	0.86 (0.35, 2.08)	0.037
	≥140	130–<140	2.17 (1.21, 3.88)	<.001	≥7.0	6.5–<7.0	3.17 (1.53, 6.56)	<0.001

^†^The logistic model is performed with the subgroup covariate as the factor, and CIs are Wald CIs. OR (OR < 1) favors alternative level. ^‡^*P* values are based on Wald chi-square tests.

## Data Availability

The data used to support the findings of this study are included within the article.
